# RNA Sequencing of Cardiac in a Rat Model Uncovers Potential Target LncRNA of Diabetic Cardiomyopathy

**DOI:** 10.3389/fgene.2022.848364

**Published:** 2022-04-13

**Authors:** Yangbo Xi, Dongping Chen, Zhihui Dong, Hingcheung Lam, Jiading He, Keyi Du, Can Chen, Jun Guo, Jianmin Xiao

**Affiliations:** ^1^ Department of The First Clinical Medical College, Jinan University, Guangzhou, China; ^2^ Department of Cardiology, The First Affiliated Hospital of Jinan University, Guangzhou, China; ^3^ Central Laboratory, The Dongguan Affiliated Hospital of Jinan University, Binhaiwan Central Hospital of Dongguan, Dongguan, China; ^4^ Department of Pathology, The Dongguan Affiliated Hospital of Jinan University, Binhaiwan Central Hospital of Dongguan, Dongguan, China; ^5^ Department of Cardiology, The Dongguan Affiliated Hospital of Jinan University, Binhaiwan Central Hospital of Dongguan, Dongguan, China

**Keywords:** diabetic cardiomyopathy, long non-coding RNA, transcriptome sequencing, bioinformatic analyses, ceRNA

## Abstract

**Background:** Diabetic cardiomyopathy (DCM) is one of the major causes of heart failure in diabetic patients; however, its pathogenesis remains unclear. Long non-coding RNAs (lncRNAs) are involved in the development of various cardiovascular diseases, but little is known in DCM.

**Objective:** The present study was conducted to investigate the altered expression signature of lncRNAs and mRNAs by RNA-sequencing and uncovers the potential targets of DCM.

**Methods:** A DCM rat model was established, and the genome-wide expression profile of cardiac lncRNAs and mRNAs was investigated in the rat model with and without DCM by RNA-sequencing. Bioinformatics analysis included the co-expression, competitive endogenous RNA (ceRNA) network, and functional enrichment analysis of deregulated lncRNAs and mRNAs.

**Results:** A total of 355 lncRNA transcripts and 828 mRNA transcripts were aberrantly expressed. The ceRNA network showed that lncRNA XR_351927.3, ENSRNOT00000089581, XR_597359.2, XR_591602.2, and XR_001842089.1 are associated with the greatest number of differentially expressed mRNAs and AURKB, MELK, and CDK1 may be the potential regulatory targets of these lncRNAs. Functional analysis showed that these five lncRNAs are closely associated with fibration, cell proliferation, and energy metabolism of cardiac myocytes, indicating that these core lncRNAs have high significance in DCM.

**Conclusions:** The present study profiled the DCM-specific lncRNAs and mRNAs, constructed the lncRNA-related ceRNA regulatory network, and identified the potential prognostic biomarkers, which provided new insights into the pathogenesis of DCM.

## Introduction

Diabetic cardiomyopathy (DCM) is a diabetes mellitus (DM)–induced pathophysiological condition characterized by abnormal cardiac function and structure in the absence of hypertension, coronary artery disease, and valvular heart disease (Ritchie and Abel, 2020) and is one of the major causes of heart failure in diabetic patients. Reportedly, the prevalence of cardiac dysfunction in patients with type 1 diabetes mellitus (T1DM) and T2DM is 14.5 and 35%, respectively (Bouthoorn et al., 2018; Tan et al., 2020). Although the number of studies on DCM increased exponentially over the past decade, and the pathogenesis of this condition is yet unclear.

Accumulating evidence has shown that long non-coding RNAs (lncRNAs) play critical roles in the pathology and physiology of cardiovascular diseases (CVDs) and can be used as potential targets for the diagnosis and prevention of CVDs (Uchida and Dimmeler, 2015). LncRNAs are endogenous RNA transcripts longer than 200 nucleotides that do not have protein-coding potential. However, they are significant molecules in almost every gene function and regulation level, including cell proliferation, epigenetic regulation, and genomic imprinting (Wang and Sun, 2020). Some studies indicated that lncRNAs are potential regulators of various CVDs due to their function in cardiomyocyte proliferation, differentiation, cardiac gene expression, and cardiac remodeling (Ritchie and Abel, 2020).

LncRNAs have an mRNA-like structure with a 5′-end methylated cap and a 3′-end poly-A tail and act as competitive endogenous RNAs (ceRNAs) to regulate mRNA expression by interaction with the shared miRNAs on target genes (Salmena et al., 2011). This participates in the occurrence and development of various CVDs (Li et al., 2019), including congenital heart disease (Wang and Yuan, 2019; Zhang et al., 2021), cardiac hypertrophy (Viereck et al., 2020), heart failure (Li et al., 2019), and cardiac fibrosis (Hao et al., 2019) by epigenetic regulation of target genes. However, few studies have focused on the role of lncRNAs in DCM development, and the lncRNA-related ceRNA regulation in DCM is yet to be clarified.

DCM occurs as a result of hyperglycemia-induced impairment of myocardial function. A streptozocin (STZ)-induced rat model of T1DM has been generated to study the impact of diabetes on the heart. In the present study, we established the DCM rat model and investigated the altered expression signature of lncRNAs and mRNAs by RNA-sequencing. Furthermore, we conducted bioinformatics analysis of the deregulated lncRNA–mRNA with co-expression, ceRNA network, and functional enrichment analysis. The parallel analysis of lncRNA and mRNA expression profiles allowed us to evaluate the impact of lncRNA deregulation and their potential pathogenetic role in DCM.

## Methods

### Establishment of the Animal Model and Echocardiographic Analysis

The animal study was reviewed and approved by the Medical Ethics Committee of the Dongguan Affiliated Hospital of Jinan University. After overnight fasting, the model group rats (8 weeks old, male, *n* = 25) were injected a single dose of streptozocin (STZ) solution (1% in citrate saline, freshly prepared, 50 mg/kg, intraperitoneal (i.p.)). The control group rats (8 weeks old, male, *n* = 12) were injected (i.p.) with equivalent doses of citrate saline (STZ solvent) and fed under the same conditions. Three rats were housed together in a cage and given adequate water and standard rat chow. The blood glucose level was measured 5 days after i.p. injection. The rats with blood glucose >16.7 mmol/L for two consecutive days were presumed to be diabetic.

For echocardiographic analysis, the rats were anesthetized with 2% (vol/vol), 50 mg/kg phenobarbital (H20057384), and echocardiography was performed after 20 weeks post-intraperitoneal injection. The left ventricle internal dimension at end-diastole (LVID;d), left ventricle internal dimension at end-systole (LVID;s), left ventricle posterior wall thickness at end-diastole (LVPW;d), and interventricular septum thickness at end-diastole (IVS;d) were measured by M-mode tracing using an L15-7io probe (Ultrasound Transducer Bothell, WA, United States) (*n* = 11 in the control group, *n* = 25 in the model group). The percentage of fractional shortening (FS) was calculated as follows: [(LVEDD-LVESD)/LVEDD] × 100 and ejection fraction (EF) percentage using the equation: [(EDV−ESV)/EDV)] × 100, where EDV represents end-diastolic volume and ESV represents end-systolic volume.

### Estimation of Histological and Blood Parameters

After administering anesthesia with 70 mg/kg phenobarbital, thoracotomy was performed to collect blood by puncturing with a syringe needle in the left ventricle. The blood insulin, glucagon, total cholesterol (TC), triglyceride (TG), high-density lipoprotein cholesterol (HDL-c), low-density lipoprotein cholesterol (LDL-c), brain natriuretic peptide (BNP), cardiac troponin I (cTn I), and creatinine (Cr) were measured using assay kits (Nanjing Jiancheng Bio.: A111-A113-2-1, CUSABIO: CSB-E07972r, CSB-E08594r, CSB-E05070r, CSB-E12800r, China). (*n* = 4 in the control group, *n* = 5 in the model group).

After overdosing with anesthesia, the myocardium from the left ventricles of the rats was harvested (*n* = 5 for each group). For histological analysis, the freshly harvested myocardium samples were fixed with 4% paraformaldehyde, embedded in paraffin, sectioned into 4-μm-thick slices, and stained with hematoxylin and eosin (H&E, BBC Biochemical) and Masson’s trichome staining (Abcam, ab150681). To further analyze the ultrastructural changes in the cardiomyocytes, transmission electron microscopy analysis was conducted at the Guangzhou Huiyuanyuan Pharmaceutical Technology Co., Ltd. For this, the myocardium samples were fixed with 2.5% glutaraldehyde and 1% osmium acid, rinsed with 0.1 M phosphate buffer, embedded in paraffin, sectioned into 50–70 nm thick slices, and observed under a transmission electron microscope (Japan Electron Optics Laboratory Co., Ltd., JEM-1400 PLUS).

### Tissue Collection and RNA-Sequencing

For RNA-sequencing, the myocardium harvested from the left ventricle of rats was immediately snap-frozen at −80°C before RNA extraction. Total RNA was extracted from heart samples using the mirVana miRNA Isolation Kit (Ambion) following the manufacturer’s protocol. RNA integrity was evaluated using the Agilent 2100 Bioanalyzer (Agilent Technologies, Santa Clara, CA, United States). The samples with RNA integrity number (RIN) ≥7 were subjected to subsequent analysis. The libraries were constructed using TruSeq Stranded Total RNA with Ribo-Zero Gold, according to the manufacturer’s instructions, sequenced on the Illumina sequencing platform (HiSeqTM 2500), and 150-bp/125-bp paired-end reads were generated. The RNA-depleted RNA-seq was carried out at the laboratory of Shanghai OE Biotech Company.

### RNA-Sequencing Data Validation by RT-qPCR

To validate the expression profile data obtained from RNA-sequencing, we selected eight lncRNAs and eight mRNAs that met the screening criteria for validation using RT-qPCR. Total RNA was isolated from five samples each from the control and model groups. An equivalent of 1 μg RNA was converted to complementary DNA (cDNA) as per the manufacturer’s guidelines (Takara, RR047Q, Japan). The expression level of lncRNA and mRNA was determined by RT-qPCR using Universal SYBR qPCR Master Mix (Biosharp, BL697A, China). PCR was performed on an SLAN-96S instrument (Shanghai Hongshi Medical Technology Co., Ltd., China) using a PrimeScript^™^ RT reagent Kit (Takara, RR047Q, Japan) and the reaction conditions were as follows: pre-denaturation at 95°C for 3 min, 40 cycles of denaturation at 95°C for 5 s, and annealing at 60°C for 1 min. The PCR reaction consisted of 10.0 μl of SYBR Mix, 1.0 μl of PCR forward primer, 1.0 μl of PCR reverse primer, 1.0 μl of the cDNA template, and 7.0 μl of RNase-free dH_2_O in a total volume of 20 μl. *GAPDH* was used as the internal reference for real-time PCR. The relative expression of the target gene was calculated using the 2^−ΔΔCt^ method. The primers are listed in Table 1.

**TABLE 1:** List of primer sequences for RT-qPCR.

	Forward (5′-3′)	Reverse (5′-3′)
mRNA		
Bok	5′-CCC​AGC​GTA​TAT​CGG​AAT​GTG​G-3′	5′-CAC​TAC​CTT​GCC​CCA​TGT​GA-3′
Hmox1	5′-CTT​CCC​GAG​CAT​CGA​CAA​CC-3′	5′-AAT​GTT​GAG​CAG​GAA​GGC​GG-3′
Ckb	5′-GAC​GTT​CCT​GGT​GTG​GAT​CA-3′	5′-GAG​TGA​GGC​CAG​TGC​AGA​AT-3′
Pla2g7	5′-GTT​CCA​AGG​CTC​TCA​GTG​CAA-3′	5′-CTC​ACG​GGA​AAC​ATC​CAC​GG-3′
Col1a2	5′-TAC​AAC​GCA​GAA​GGG​GTG​TC-3′	5′-TCC​AGG​TAC​GCA​ATG​CTG​TT-3′
Col3a1	5′-CCC​TGA​ACT​CAA​GAG​CGG​AGA-3′	5′-ACC​AGC​ATC​TGT​CCA​CCA​GT-3′
Eno3	5′-ATC​AGT​GGG​GAG​AAG​CTC​GG-3′	5′-CCC​AGC​CAT​TAG​ACT​GTG​CC-3′
Hmox1	5′-CTT​CCC​GAG​CAT​CGA​CAA​CC-3′	5′-AAT​GTT​GAG​CAG​GAA​GGC​GG-3′
GAPDH	5′-ACC​ACC​ATG​GAG​AAG​GCT​GC-3′	5′-CTC​AGT​GTA​GCC​CAG​GAT​GC-3′
lncRNA		
XR_001842342.1	5′-TTC​TTG​CCC​CTC​CTT​CCT​AGT-3′	5′-GGA​ACA​TCA​GCG​GAG​ACC​CT-3′
XR_590344.2	5′-TGG​AAG​AAG​AGG​GCC​ACC​AA-3′	5′-CAG​ATC​AGG​CTG​ACG​GCA​AG-3′
XR_357664.3	5′-ACG​AGA​TAA​GCC​GGA​TGC​AAG-3′	5′-CGG​GTG​CAA​AGT​GTA​GTG​GT-3′
XR_350940.2	5′-AAA​GTG​TTC​TTG​CCC​CTC​CTT-3′	5′-AGA​CCG​TCA​ACA​GCT​TAG​CC-3′
XR_349856.3	5′-TAT​ACA​CAT​TGC​GGG​GCC​AAC-3′	5′-AGA​AAT​GCC​CAC​AGC​AGT​AGT-3′
XR_146366.4	5′-GCA​GCC​AGA​AGC​AAA​TGA​GC-3′	5′-GAC​CAA​GCA​CCA​GCT​ATG​GG-3′
XR_001842089.1	5′-TTG​GCT​GGT​TTT​TCT​GGG​CAT-3′	5′-ACC​AAA​CCC​CAG​CAT​ATC​GG-3′
ENSRNOT80276	5′-GGA​GCA​CTG​CCC​TGG​TTA​GA-3′	5′-CTC​GTT​TCT​CGT​GGG​CGT​TC-3′

### Bioinformatic Analysis

The raw reads generated during high-throughput sequencing are in the FASTQ format. In order to obtain high-quality reads for subsequent analysis, the raw reads were subjected to a quality filter. Trimmomatic (Bolger et al., 2014) was first used for adapter removal, and then low-quality bases and N-bases or low-quality reads were filtered out. Using HISAT2 (Kim et al., 2015) to align clean reads to the reference genome of the experimental species, the sample was assessed by genomic and gene alignment. Stringtie software (Pertea et al., 2015) was utilized to assemble the reads, and the new transcript was spliced. Then, the candidate lncRNA transcripts were selected by comparing the gene annotation information of the reference sequence produced by Cuffcompare (Trapnell et al., 2012) software. Finally, transcripts with coding potential were screened out by CPC (Kong et al., 2007), Pfam (Finn et al., 2006), and PLEK (Li et al., 2014) to obtain lncRNA-predicted sequences.

Subsequently, Size Factors function of the DESeq (2012) R package was used to normalize the counts, and nbinom test function was applied to calculate the *p*-value and fold-change values for the comparison of differences. The differential transcripts with *p*-values ≤ 0.05 and fold change ≥ 2 were selected to identify the differentially expressed lncRNAs and mRNAs. Kyoto Encyclopedia of Genes and Genomes (KEGG) pathway enrichment and Gene Ontology (GO) analysis of differentially expressed mRNAs (DE-mRNAs) were conducted using the SWISS-PROT database (http://www.gpmaw.com) and online analysis tool KAAS (http://www.genome.jp/tools/kaas/).

### Construction of the ceRNA Network

In order to construct the ceRNA regulatory network, the miRNA data (FASTA file) rno_miRNA.fa were obtained from the miRBase platform (www.mirbase.org).The miRNA–mRNA and lncRNA–miRNA interactions were predictive analyses by miRanda (v3.3a). The ceRNA score was calculated using the MuTaME (H. Wang et al., 2020), and the *p*-value of the ceRNA interactions was based on the hypergeometric distribution (Liu K. et al., 2013).

Pearson’s correlation test was used to calculate the correlation between the expression of differential lncRNA (length <6000 nt) and differential mRNA expression data: the correlation of the pair was >0.8 or < −0.8 and *p* < 0.05. The positively related mRNA–lncRNA co-expression interactions were screened out. The ceRNA network was based on the intersection interactions between the co-expression and ceRNA score. The lncRNA–miRNA–mRNA (ceRNA) interaction regulatory network was integrated using Cytoscape software (v3.7.2).

## Results

### General Characteristics and Echocardiography of Animals

**FIGURE 1:**
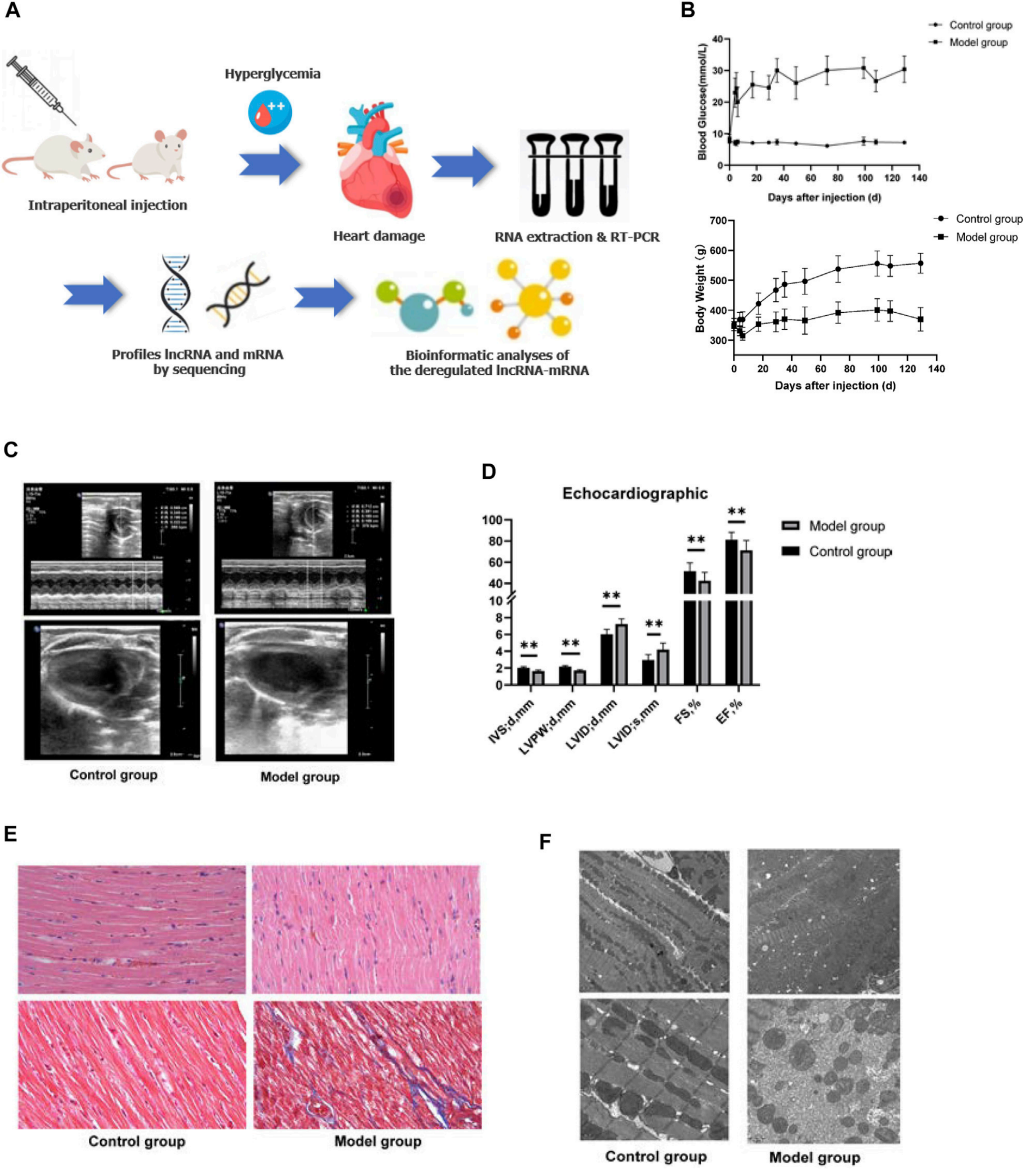
General characteristics of the animal model. **(A)** Schematic depiction of the experimental design. **(B)** Blood glucose and body weight of the animals in the two groups after STZ injection. **(C**,**D)** Echocardiography of the animal model. Left ventricle internal dimension at end-diastole (LVID;d), left ventricle internal dimension at end-systole (LVID;s), ejection fraction (EF), fractional shortening (FS), left ventricle posterior wall thickness at end-diastole (LVPW;d), interventricular septum thickness at end-diastole (IVS;d). Data are represented as mean ± SEM, **p < 0.05. **(E)** HE (upper part), and Masson’s (lower part) staining. Masson’s staining identified massive fibrosis in the diabetic myocardium. **(F)** Transmission electron microscopy observed that cardiomyocytes in diabetic hearts appear damaged with loss of sarcomeres and pathological changes in the mitochondrial shape involving loss or reorientation of cristae and matrix depletion.

The plasma and echocardiographic parameters of the animal are shown in Figure 1 and Table 2. The rats of the control group lost weight at 1 week after the STZ injection, and the body weight was significantly lesser than that of the age-matched rats of the control group up to 20 weeks of follow-up (*p* < 0.05). The blood glucose level of the model group rats increased significantly within 1 week after the STZ injection compared to the control group and remained at a higher level (>16.7 mmol/L) for up to 20 weeks post follow-up (Figures 1A,B). The histological analysis revealed that the spaces between cardiomyocytes in diabetic hearts are enlarged with disorders of myocardial cell arrangement compared to control hearts, indicating altered cellular structure. Masson’s staining showed massive fibrosis in the diabetic myocardium (Figure 1E). At the ultrastructural level, cardiomyocytes in diabetic hearts were damaged with the loss of sarcomeres, and the pathological changes in the mitochondrial shape involved loss or reorientation of cristae and matrix depletion (Figure 1F). Furthermore, the level of BNP was substantially elevated in blood from 20-week diabetic rats compared to that in the control hearts (*p* < 0.05), indicating hyperglycemic damage to the myocardium. Simultaneously, no significant differences were detected in HDL-c, LDL-c, TG, TC, and Cr levels among the groups (Table 2). The blood level of insulin and glucagon decreased significantly in the model group, indicating impaired islet function after intraperitoneal injection of STZ.

**TABLE 2:** Plasma parameters of animals.

	Control group	Model group	*p*-value
TC, mmol/L	1.70 ± 0.14	1.54 ± 0.16	0.610
TG, mmol/L	1.23 ± 0.26	0.83 ± 0.37	0.112
LDL-c, mmol/L	0.99 ± 0.26	0.79 ± 0.39	0.394
HDL-c, mmol/L	1.48 ± 0.25	1.59 ± 0.26	0.532
Cr, μmol/L	60.08 ± 31.58	32.80 ± 9.64	0.106
BNP, pg/ml	1129.65 ± 472.17	4356.79 ± 2112.40	0.021
cTn I, pg/ml	60.83 ± 12.91	54.55 ± 7.17	0.382
Insulin, nUI/ml	901.61 ± 49.80	320.66 ± 113.43	<0.001
Glucagon, pg/ml	52.86 ± 9.84	26.19 ± 18.01	0.033

TC, total cholesterol; TG, triglyceride; LDL-c, low-density lipoprotein cholesterol; HDL-c, high-density lipoprotein cholesterol; Cr, creatinine; BNP, brain natriuretic peptide; cTn I, cardiac troponin I, data are represented as mean ± SEM.

The assessment of cardiac contractile function revealed that the echocardiogram analysis of the control and model group had significantly increased LVID and left ventricular volume (LVEV) at both end-diastole and end-systole, while IVS, LVPW, FS, and EF were significantly decreased in the model group at 20 weeks compared to those of the age-mated control group (*p* < 0.05). These data suggested that hyperglycemia causes cardiac dysfunction and cardiomyopathy (Figures 1C,D).

### LncRNA and mRNA Expression Profile in the Rat Model

**FIGURE 2:**
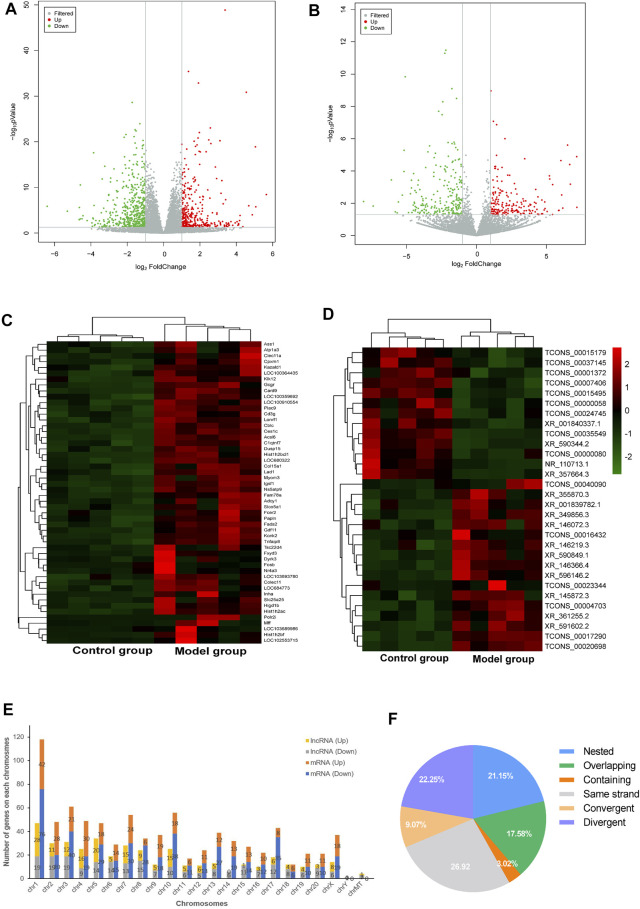
LncRNA and mRNA expression profile of the rat myocardium. **(A**,**B)** Volcano plots show DE-mRNAs **(A)** and lncRNAs **(B)** in the two groups. The red and green points represent up- and downregulated mRNAs/lncRNAs (compare to the control group), respectively. The horizontal green line depicts *p < 0.05, whereas the vertical green line shows a two-fold change in up and downregulation. **(C**,**D)** Hierarchical clustering analysis presents differentially expressed mRNAs **(C)** and lncRNAs **(D)** and red and green represent up- and down regulated mRNAs/lncRNAs with ≥ two-fold changed, respectively (3 < FPKM<50). **(E)** Chromosomal distribution of deregulated mRNAs and lncRNAs. **(F)** Intergenic lncRNAs included convergent, divergent, and same strand accounting for 58% and genic lncRNAs included containing, nested, and overlapping were accounted for 42%.

In order to uncover the deregulated genes and lncRNA expression, RNA-sequencing on the myocardium from both DCM rats (*n* = 5) and normal rats (*n* = 5) was performed. The lncRNA/mRNA expression profile in the rat myocardium is shown in Figures 2A–D. Among 22,601 mRNAs and 15,633 lncRNAs transcripts, 339 mRNAs and 182 lncRNAs were upregulated and 489 mRNAs and 227 lncRNAs were downregulated in the model group (fold change >2.0 and *p* < 0.05) (Supplementary Table S1). In addition, the hierarchical clustering analysis revealed that the regulatory profiles of mRNAs and lncRNAs differed significantly between the DCM rats compared to the controls (Figures 2C,D).

The chromosomal distribution of deregulated lncRNAs is shown in Figure 2E. Chromosome 1 had the maximum number of deregulated lncRNAs and mRNAs. The classification of differently expressed lncRNAs in the rat myocardium of the two groups is shown in Figure 2F. Intergenic and genic lncRNAs accounted for 58 and 42% of differently expressed lncRNAs in the DCM rat myocardium, respectively.

### Validation of lncRNAs and mRNAs by RT-qPCR Analysis

**FIGURE 3:**
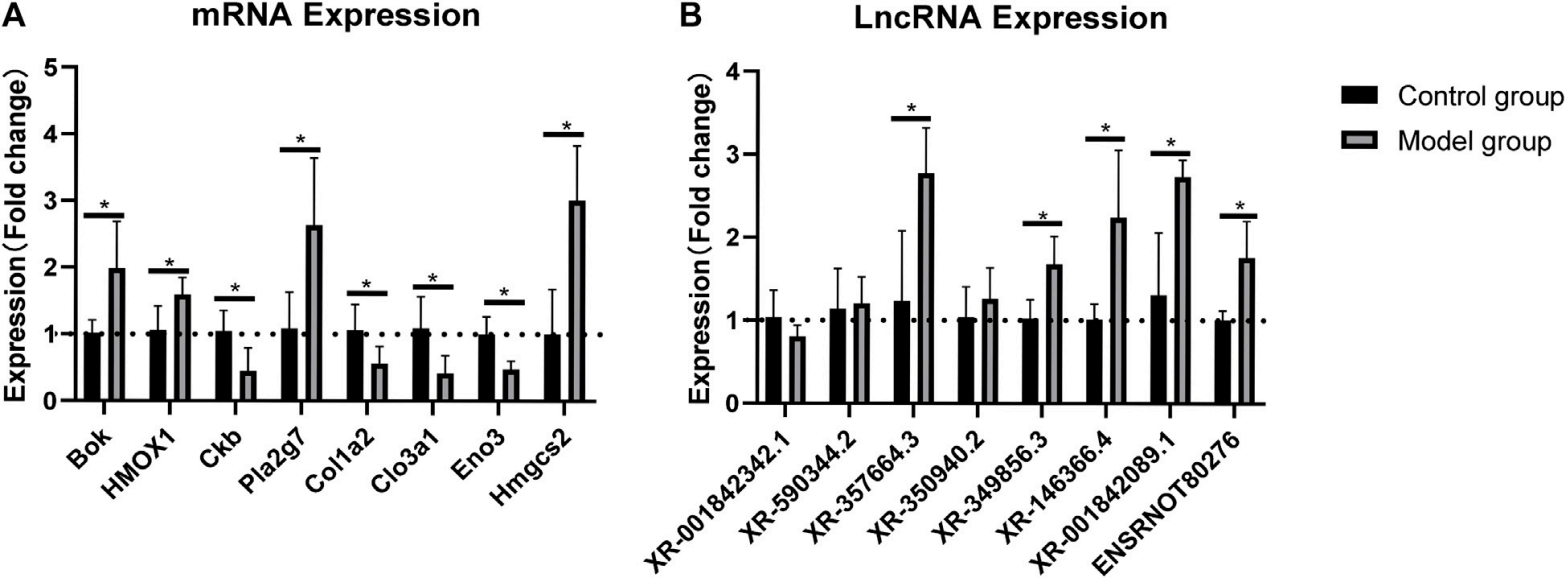
Validation of lncRNAs and mRNAs by RT-qPCR analysis. Relative expression of each lncRNA and mRNA was normalized to that of *GAPDH*. Data are represented as mean ± SEM, *p < 0.05.

The RNA-sequencing data were validated by RT-qPCR. The results showed that the expression levels of the mRNAs *Bok*, *HMOX1*, *Pla2g7*, and *Hmgcs2* were upregulated, whereas those of *Ckb*, *Clo1a2*, *Clo3a1*, and *Eno* were downregulated in the rat myocardium (Figure 3A). Similarly, the expression levels of the lncRNAs XR_357664.3, XR_350940.2, XR_349856.3, XR_146366.4, XR_001842089, and ensrnot80276 were upregulated, while those of XR_3001842342.1 and XR_590344.2 were downregulated in the rat myocardium (Figure 3B). The RT-qPCR results were consistent with the RNA-sequencing data in both lncRNAs and mRNAs.

### Functional Enrichment Analysis of DE-mRNAs

**FIGURE 4:**
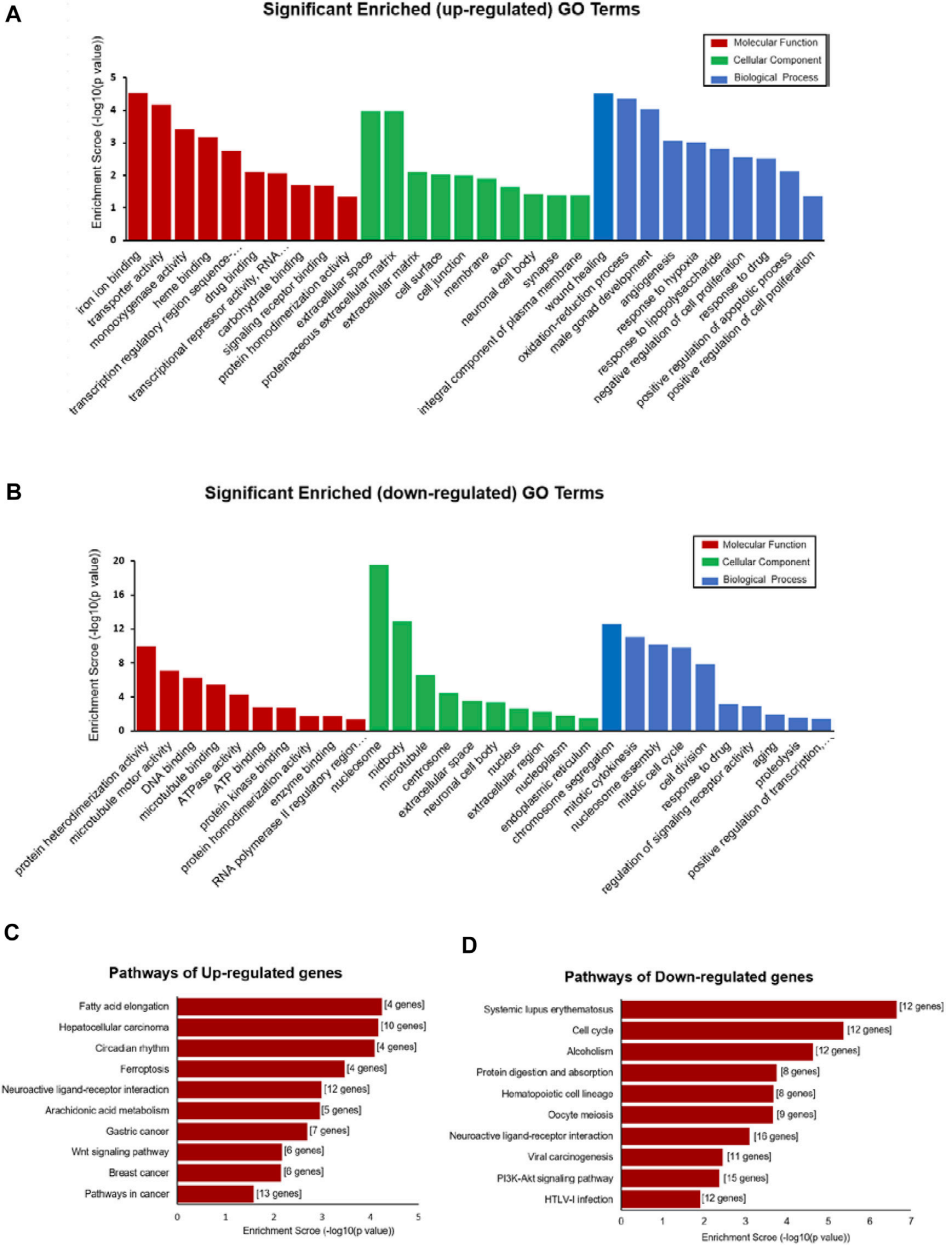
Functional enrichment analysis of DE-mRNAs. **(A**,**B)** Top 30 GO terms (comprises cellular component (CC), molecular function (MF), and biological process (BP)) consisting of significantly enriched upregulated GO terms **(A)** and downregulated GO terms **(B)**. **(C**,**D)** Pathways depict the significant difference among the control and DCM groups, significantly upregulated pathway terms, **(C)** and downregulated pathway terms **(D)**.

GO and KEGG pathway analyses were performed to determine the potential function of DE-mRNA. GO enrichment analysis consists of three levels: molecular function, biological process, and cellular component; each one explains the biological function of the genes at different levels. The results showed that the upregulated mRNAs were associated with regulation of wound healing (GO:0042060), oxidation–reduction process (GO:0055114), angiogenesis (GO:0001525), response to hypoxia (GO:0001666), and regulation of apoptotic process (GO:0008284 and GO:0008285) (Figure 4A), while the downregulated mRNAs were associated with cell division processes such as chromosome segregation (GO:0007059), mitotic cytokinesis (GO:0000281 and GO:0000278), and cell division (GO:0051301) (Figure 4B) at the biological process level. In the category of cellular component, the upregulated mRNAs were enriched in the extracellular matrix (GO:0031012 and GO:0005615) and membrane (GO:0016020) (Figure 4A), while the down deregulated mRNAs were associated with the nucleus (GO:0005634) and nucleoplasm (GO:0005654) (Figure 4B). For molecular function, the deregulated mRNAs were related to protein homodimerization activity (GO:0042803), iron ion binding (GO:0005506), DNA binding (GO:0003677), and ATP binding (GO:0005524) (Figures 4A,B) (Supplementary Table S2).

KEGG pathway enrichment analysis is used for functional annotation in order to elucidate the related functions and pathways of the differentially expressed genes. Our KEGG pathway analysis results showed that the DE-mRNAs were associated with the PI3K-Akt signaling pathway (rno05166), viral carcinogenesis (rno05203), cell cycle (rno04110), and alcoholism (rno05034) (Figure 4D). The upregulated mRNA enrichment pathways included cancer (rno05200), Wnt signaling pathway (rno04310), fatty acid elongation (rno00062), and ferroptosis (rno04216) (Figure 4C and Supplementary Table S2). The results suggested a major role of those pathways in the occurrence and development of DCM.

### LncRNA–mRNA Co-expression and ceRNA Regulatory Network

**FIGURE 5:**
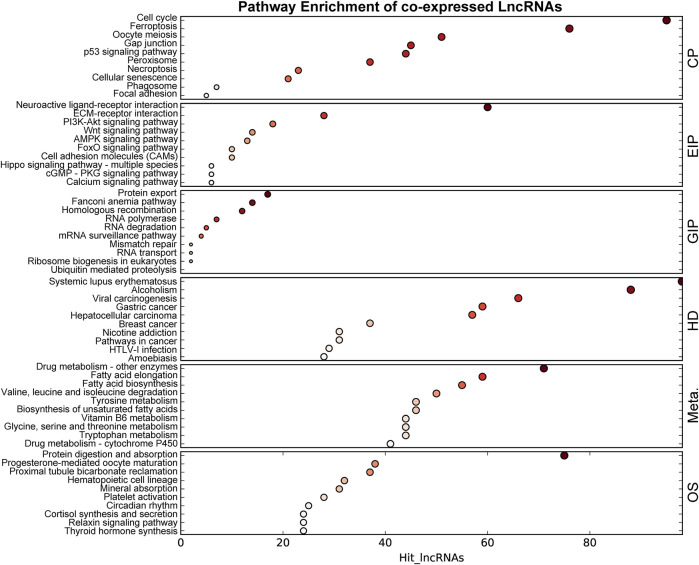
KEGG enrichment of genes in the co-expressed network. The size of the bubble represents the number of differential genes, and the bubble’s color represents enriched *p*-value. Hit_lncRNAs indicate the number of lncRNAs with co-expressed mRNAs. KEGG level 1 category included cellular processes (CP), environmental information processing (EIP), genetic information processing (GIP), human diseases (HD), metabolism (Meta.), and organismal systems (OS).

The co-expression networks (CENs) are constructed based on the evaluation of the co-expression correlation between genes and lncRNAs according to the normalized intensity of the signal values. The CENs are commonly used to reveal the core regulatory lncRNAs. The significantly co-expressed lncRNAs–mRNAs (Pearson’s correlation >0.8 and *p* < 0.05) were integrated into CENs. Finally, 29,289 connections were identified between 385 lncRNAs and 827 mRNAs in the DCM rat myocardium (Supplementary Table S3). The top 10 KEGG level 1 enriched genes in the CENs are shown in Figure 5. KEGG level 1 category included cellular processes (CP), environmental information processing (EIP), genetic information processing (GIP), human diseases (HD), metabolism (Meta.), and organismal systems (OS).

**FIGURE 6:**
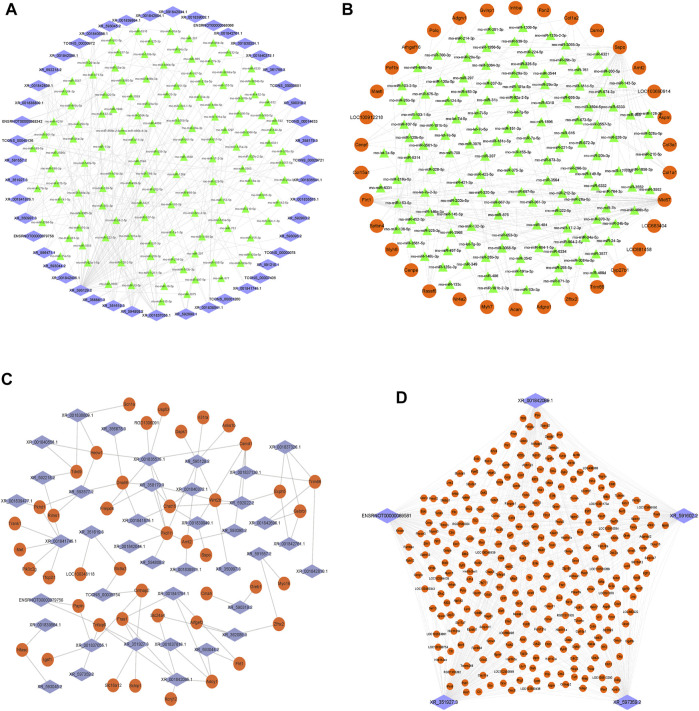
Construction of the lncRNA–miRNA–mRNA network. **(A)** Network of the top 200 miRNA–lncRNA interactions. **(B)** Network of the top 200 miRNA–mRNA interactions. **(C)** Network of the top 100 lncRNA–mRNA of ceRNA interactions. **(D)** Top five lncRNA XR_351927.3, ENSRNOT00000089581, XR_597359.2, XR_591602.2, and XR_001842089.1 connections with DE-mRNAs in the ceRNA network. The blue diamond represents lncRNAs, the green triangle represents miRNAs, and the orange circle represents mRNAs.

Next, we constructed the ceRNA regulatory networks based on the lncRNA–mRNA CENs. A total of 206,942 pairs of miRNA–mRNA interactions and 66,471 pairs of lncRNA–miRNA interactions were obtained. Then, the ceRNA network was constructed by integrating these interactions using Cytoscape software v3.7.2 (Figure 6). The top 200 miRNA–mRNA and lncRNA–miRNA and top 100 lncRNA–mRNA of the ceRNA interaction network are shown in Figures 6A–C and Supplementary Table S3.

Among them, the lncRNA, XR_351927.3, ENSRNOT00000089581, XR_597359.2, XR_591602.2, and XR_001842089.1 have maximum connections with DE-mRNAs in the ceRNA network, indicating that these lncRNAs might comprise a significant core (Figure 6D and Supplementary Table S3). Bioinformatics analysis revealed that these mRNAs related to the five lncRNAs are closely associated with fibration and energy failure. Some of these molecules were ARG1 and SERPINH1 related to collagen biosynthetic process (GO:0032964); GREM1, COL1A2, LUM, SERPINH1, and SCX related to collagen fibril organization (GO:0030199); MELK, UHRF1, CKS2, CDK1, MKI67, FAM83D, CD34, AURKB, E2F8, and BOK related to cell proliferation (GO:0008283); TOP2A, KIF14, ATP1A3, BUB1B, TTK, KIF11, AURKB, P2RY1, PBK, NEK2, CKB, EPHB1, POLQ, UBE2C, PLK1, ACSL6, KIF24, KIF23, NEK2L1, KIF22, MASTL, ASS1, MYO16, CIT, SBK2, CENPE, MELK, KIF18B, KIFC1, KIF4A, CDK1, TUT1, KIF20A, KIF20B, FKBP4, MYH6, and TTLL9 related to ATP binding (GO:0005524). The deregulated mRNAs, *AURKB*, *MELK*, and *CDK1*, repeated multiple enrichment in related items might be associated with the pathogenesis of DCM.

## Discussion

Previous studies indicated that diabetes has adverse effects on the different cell types of the heart, including endothelial cells, fibroblasts, and cardiomyocytes. DCM is attributed to hyperglycemia-induced impairment of myocardial function, and heart failure is the endpoint of DCM. In the present study, we used STZ to induce diabetes and established a DCM animal model (Liu Z.-W. et al., 2013; Riehle and Bauersachs, 2018). STZ is a glucosamine-nitrosourea similar to the glucose molecule that needs to be transported into the cells alone by the low-affinity glucose transporter (GLUT2) on islet β-cells, destroying the islet β-cells, resulting in insulin synthesis, decreased secretion, and disrupted glucose metabolism; currently, it is the most widely used chemical inducer in diabetic animal models (Bonnevie-Nielsen et al., 1981). The present study confirmed that STZ-induced rats exhibit decreased body weight and plasma insulin levels. Furthermore, our rat model also confirmed that STZ-induced diabetes leads to a pronounced DCM characterized by myocardial fibrosis, mitochondrial dysfunction, and associated diastolic and systolic dysfunction, which is similar to that observed previously (Joffe et al., 1999; Xie et al., 2020; Yu et al., 2021). In addition, RNA-sequencing of cardiac tissue uncovers a large number of DE-lncRNAs and mRNAs, indicating that lncRNA epigenetic regulation plays a major role in heart damage during the diabetic state. Several studies have demonstrated the regulatory role of lncRNAs in various CVDs (Liu et al., 2018; Lv et al., 2018). The lncRNA can target and modulate the physiological functions of cardiomyocytes (Uchida and Dimmeler, 2015; Y.; Wang et al., 2020) and regulate them in a cell-type/tissue-specific manner (Ritchie and Abel, 2020). The role of lncRNAs in CVDs has gained increasing attention. Thus, it is essential to focus on the role of lncRNAs in DCM. Whole-transcriptome profiling of lncRNAs and mRNAs was conducted in the DCM animal model, which opened up new possibilities to explore the lncRNA dysregulation in the pathogenesis of DCM.

The RNA-sequencing results revealed significant differences in lncRNA/mRNA expression between the DCM and control groups. Next, we identified 409 lncRNAs aberrantly expressed in the myocardium of DCM rats. However, further investigation is required to identify the potential lncRNA targets for DCM progression and pathogenesis. Diabetes and CVDs are associated with genetic predisposition (Dahlstrom and Sandholm, 2017; Sandholm and Groop, 2018). These deregulated lncRNAs in this study were not evenly distributed on 21 chromosomes. As shown in Figure 2E, chromosome 1 has the maximal number of deregulated lncRNAs compared to other chromosomes, indicating that chromosome 1 may be susceptible to DCM pathology. Based on the correlation between lncRNAs and their affiliated protein-coding genes, lncRNAs are subdivided into the following types: genic lncRNA (lncRNA overlapping a protein-coding transcript at one or more nucleotides, including containing, nested, and overlapping) and intergenic lncRNA (lincRNA, lncRNA not overlapping a protein-coding transcript, including convergent, divergent, and same strand) (Ransohoff et al., 2018). The CEN results showed that 80% of lincRNAs were co-expressed with their neighboring genes, indicating that lincRNA should be studied with respect to the underlying regulatory mechanisms. The CENs indicated that 80% of lincRNAs and their adjacent genes were co-expressed in a similar direction, which could be useful to decipher the underlying regulatory mechanisms. Many aberrantly expressed lincRNAs in DCM suggest that lncRNAs may regulate the development of DCM *via* protein-coding genes.

Moreover, our results showed that the expression of several mRNAs was dysregulated in DCM. Taken together, 827 mRNAs were found to be differentially expressed in DCM. Consistent with the distribution of deregulated lncRNAs, chromosome 1 had the maximum number of DE-mRNAs. Based on the GO and KEGG analyses, upregulated genes were significantly enriched for pathways in cancer (rno05200), neuroactive ligand–receptor interaction (rno04080), fatty acid elongation (rno00062), oxidation–reduction process (GO:0055114), and cell proliferation (GO:0008285 and GO:0008284), whereas downregulated mRNAs were significantly enriched with neuroactive ligand–receptor interaction (rno04080), PI3K-Akt (rno04151), protein homodimerization activity (GO:0042803), microtubule motor activity (GO:0003777), and ATPase activity (GO:0016887). Previous studies depicted that the signaling pathways, such as fatty acid elongation, cell proliferation, and oxidation–reduction processes, are associated with DCM pathogenesis. Some studies confirmed that diabetes patients have a high prevalence of cancer, viral infections, and tuberculosis (Blumenthal et al., 2017; Desbois and Cacoub, 2017). Therefore, we speculated that these regulated mRNAs in diabetes might be associated with increased cancer and infection. Moreover, the KEGG pathway analysis revealed that the pathways, such as neuroactive ligand–receptor interaction, protein homodimerization activity, and cell proliferation, were involved in the pathogenesis of DCM. Previous studies have shown that protein homodimerization is associated with the complications of diabetes, including neuropathy (Iyer et al., 2019), diabetic foot ulcers (Embil and Nagai, 2002) and diabetic nephropathy (Erol et al., 2019). These pathway analysis results demonstrated that the phenotype of the STZ-induced DCM model is similar to that observed in diabetic progressive DCM patients, providing a new rationale for further study on DCM. In addition, the results of the ceRNA regulatory network revealed that lncRNA, XR_351927.3, ENSRNOT-00000089581, XR_597359.2, XR_591602.2, and XR_001842089.1 have maximum connections with DE-mRNAs, and AURKB, MELK, and CDK1 are potential regulatory targets of these lncRNAs throughout the development of DCM. Given that these five lncRNAs are associated with fibration, cell proliferation, and energy metabolism of cardiac myocytes, they may serve as potential therapeutic and diagnostic targets for DCM.

In conclusion, heart damage in the diabetic state is a major cause of cardiovascular complications in diabetic patients. The present study showed that many lncRNAs and mRNAs are deregulated in the DCM myocardium. In this study, an lncRNA-related ceRNA regulatory network was constructed to uncover the potential target lncRNAs, which provided therapeutic targets or diagnostic biomarkers of DCM.

## Data Availability

The datasets presented in this study can be found in online repositories. The names of the repository/repositories and accession number(s) can be found below: Gene Expression Omnibus, accession number GSE197999.
